# Improved YOLOv8 Network of Aircraft Target Recognition Based on Synthetic Aperture Radar Imaging Feature

**DOI:** 10.3390/s25103231

**Published:** 2025-05-21

**Authors:** Xing Wang, Wen Hong, Yunqing Liu, Guanyu Yan, Dongmei Hu, Qi Jing

**Affiliations:** 1 College of Electrical and Information Engineering, Changchun University of Science and Technology, Changchun 130022, China; xingwang_1983@126.com (X.W.);; 2 College of Electrical and Information Engineering, Beihua University, Jilin 132013, China; m15699554195@163.com (G.Y.);; 3 Aerospace Information Research Institute, Chinese Academy of Sciences, Beijing 100045, China

**Keywords:** SAR, YOLOv8, Shi–Tomasi, enhanced Lee, CA, swin transformer

## Abstract

The grayscale images of passenger aircraft targets obtained via Synthetic Aperture Radar (SAR) have problems such as complex airport backgrounds, significant speckle noise, and variable scales of targets. Most of the existing deep learning-based target recognition algorithms for SAR images are transferred from optical images, and it is difficult for them to extract the multi-dimensional features of targets comprehensively. To overcome these challenges, we proposed three enhanced methods for interpreting aircraft targets based on YOLOv8. First, we employed the Shi–Tomasi corner detection algorithm and the Enhanced Lee filtering algorithm to convert grayscale images into RGB images, thereby improving detection accuracy and efficiency. Second, we augmented the YOLOv8 model with an additional detection branch, which includes a detection head featuring the Coordinate Attention (CA) mechanism. This enhancement boosts the model’s capability to detect small and multi-scale aircraft targets. Third, we integrated the Swin Transformer mechanism into the YOLOv8 backbone, forming the C2f-SWTran module that better captures long-range dependencies in the feature map. We applied these improvements to two datasets: the ISPRS-SAR-aircraft dataset and the SAR-Aircraft-1.0 dataset. The experimental results demonstrated that our methods increased the mean Average Precision (*mAP*_50~95_) by 2.4% and 3.4% over the YOLOv8 baseline, showing competitive advantages over other deep learning-based object detection algorithms.

## 1. Introduction

Spaceborne Synthetic Aperture Radar (SAR) captures ground imagery by transmitting and receiving electromagnetic waves [1]. It can meticulously record details of ground targets and produce high-resolution images by employing synthetic radar pulse technology. In the field of Synthetic Aperture Radar (SAR) imaging, passenger aircraft are important targets of great research value. Leveraging this technology enables real-time monitoring of passenger aircraft positions and dynamics. This not only significantly bolsters aviation management safety but also substantially improves operational efficiency [2,3,4,5]. The traditional SAR image interpretation process comprises three stages: target detection, identification, and recognition [6,7,8,9,10]. The methods in the traditional SAR image interpretation process generally require expert intervention to adjust parameters and interpret results. Moreover, the SAR imaging process can be compromised by complex environments and clutter interference, which may lead to challenges in target interpretation, reduced generalization capabilities, and lower detection accuracy than expected.

Deep learning-based algorithms have demonstrated superior performance in image recognition [11,12,13,14,15,16]. Numerous scholars have significantly advanced SAR image target interpretation. Wang et al. proposed an adaptive and robust edge detector to optimize the YOLO structure, enhancing the interpretation accuracy of aircraft targets [17]. However, it may slow the convergence of network training. Xiao et al. introduced a novel network that effectively extracts aircraft targets’ scattering features [18], yet it lacks an end-to-end methodology. Zhang et al. developed the SFRE-Net architecture to enhance the relationship between scattering features, enriching semantic information and mitigating semantic conflicts during feature fusion [19]. Jiang et al. introduced a coordinate-aware mixed attention and spatial semantics joint-context approach to diminish background clutter effects and enhance the detection of small targets [20]. Sun et al. designed the CP-FCOS network structure based on the Anchor-Free method, which can better accomplish the task of ship detection in SAR images through pixel-by-pixel prediction [21]. Zhou et al. designed the SCNet-YOLO network structure based on symmetric convolution and integrated the attention mechanism. This enables it to effectively capture multi-scale targets, and it is particularly effective in enhancing the detection ability of small targets [22]. Sun et al. designed the BiFA-YOLO network structure based on bidirectional feature fusion and angular classification, which can effectively improve the detection ability of arbitrary-oriented ships [23].

While progress has been made in developing deep learning-based algorithms for interpreting aircraft targets in SAR images, significant challenges persist in enhancing the detection accuracy in practical settings. First, radar signal sampling and imaging mechanisms contribute to the discontinuity in image characteristics. This discontinuity compromises image quality, limits detailed information about aircraft targets, reduces the correlation between components, and impedes the detection of entire aircraft targets. Second, airport backgrounds are highly complex, with strong scattering structures such as terminal buildings, runways, and vehicles. These structures can obscure target features and increase the likelihood of false positives and missed detections. Third, the considerable variation in aircraft sizes poses a challenge for maintaining prediction accuracy across different scales, particularly when image resolution is low and small targets are easily overlooked [24,25]. In conclusion, deep learning models still struggle to extract feature information from small targets and differentiate targets from background noise. We utilized two SAR image datasets, the ISPRS-SAR-Aircraft dataset [26] and the SAR-Aircraft-1.0 dataset [27], which encompass seven categories of targets, including the Boeing 787, A330, Boeing 737-800, A320/321, ARJ21, A220, and others [28].

In this paper, we enhance the capabilities of the YOLOv8 algorithm [29,30] by addressing specific challenges associated with interpreting SAR images for aircraft target detection. Recognizing the limitations of the baseline YOLOv8 algorithm in handling SAR imagery [31,32,33,34], we introduce three innovative optimization methods aimed at improving feature expression, the detection of multi-scale targets, and the overall detection accuracy. Each method targets a specific problem area, contributing to a more robust model capable of handling the complexities of SAR image analysis. The effectiveness of these enhancements is validated through extensive experimental testing, demonstrating significant improvements in the detection accuracy of aircraft targets, particularly smaller ones. Our contributions are summarized as follows:

(1) Gray-to-RGB (GTR) conversion: We developed the GTR method to transform single-channel grayscale SAR images into three-channel RGB images. This conversion process augments the feature space available for the model, thereby reducing the tendency towards overfitting and enhancing detection accuracy.
(2) Four-scale detectors with coordinate attention (4SDC): To address the challenge of detecting aircraft targets of varying sizes in SAR images, we introduced the 4SDC approach. This method adaptively adjusts the weights of different scale detection branches, significantly reducing missed detections of small targets and improving the model’s ability to handle multi-scale targets.
(3) C2f-SWTran module integration: By incorporating the Swin Transformer mechanism into the backbone of YOLOv8, we created the C2f-SWTran module. This integration effectively captures fine-grained and global information within the imagery, leveraging a combination of self-attention and global perception mechanisms to enhance the processing of multi-scale feature maps and increase detection precision.

The structure of this paper is outlined as follows: Section 2 introduces the YOLOv8 network framework. Section 3 details three enhanced algorithms. Section 4 presents the experimental results and assesses the effectiveness of the optimization algorithms. Finally, Section 5 concludes the paper and outlines future research directions.

## 2. Preliminary: YOLOv8 Series Architecture

**Figure 1:**
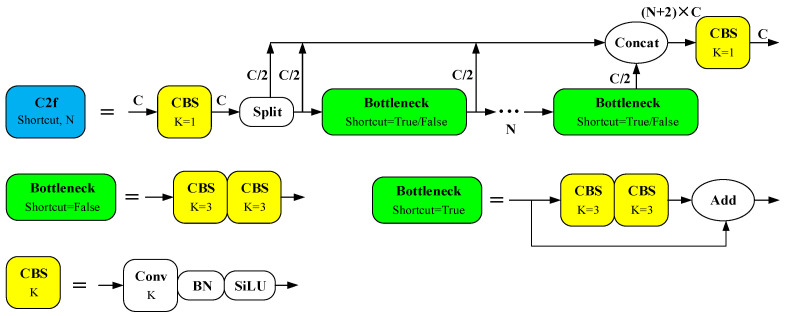
Schematic diagram of C2f network structure.

Ultralytics (Frederick, MD, USA) introduced YOLOv8 in early 2023. Compared to its predecessor YOLOv5, the YOLOv8 model retains the same architectural framework, comprising four layers: input, backbone, neck, and head. However, YOLOv8 includes enhancements in various processing details that significantly improve its overall detection performance. Specifically, in the backbone layer of YOLOv8, a Coarse-to-Fine (C2f) structure was developed, incorporating elements from the C3 residual structure of YOLOv5 and the ELAN concept from YOLOv7, as illustrated in Figure 1.

Where *C* represents the number of channels in the feature image, the number of channels in the input and output feature maps of the C2f module is *C*. *N* represents the number of repetitions of the Bottleneck module, which varies according to the chosen YOLOv8 model variant (N/S/M/L/X) and the placement of the C2f module within the network. “K” indicates the size of the convolution kernel. Within the backbone network, the Bottleneck module in the C2f setup includes a shortcut residual structure; however, this configuration is absent in the Bottleneck module of the neck network.

**Figure 2:**
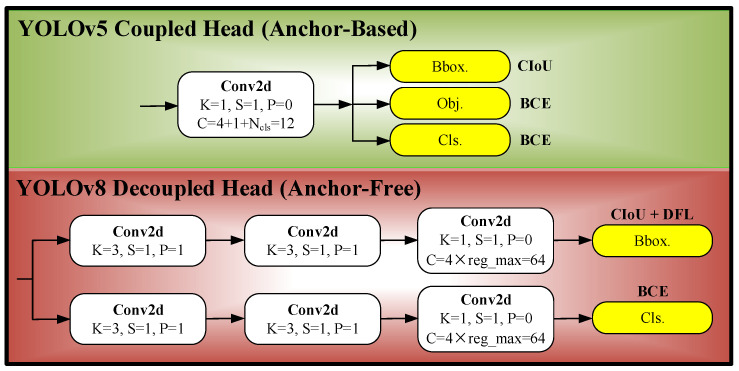
Detection head comparison of YOLOv5 and YOLOv8.

The C2f strategy divides the detection task into two phases via a split operation: rough detection and detailed analysis. Initially, the algorithm quickly locates potential targets and conducts concentrated analysis in these areas. Depending on the requirements of different scenarios, the weights between the two phases can be adaptively adjusted to balance speed and accuracy. C2f incorporates additional residual structures, facilitating freer information interaction across various network layers. These structures efficiently process and integrate information, effectively mitigating the issue of vanishing gradients often encountered in deeper networks. Unlike C3, C2f eliminates convolution operations in its branches, simplifies the model’s structure, and reduces parameter count, which decreases the risk of overfitting and enhances generalization capabilities. This configuration allows C2f to capture richer gradient flow information while maintaining lightweight model parameters and enhancing the overall detection performance of the YOLOv8 algorithm. Compared with YOLOv5, the YOLOv8 detection head employs a decoupled processing approach, discarding the object branch while retaining the classification and regression branches, and shifting from an Anchor-Based to an Anchor-Free configuration. These changes are depicted in Figure 2.

YOLOv5 integrates regression, classification, and confidence detection into a single network architecture for simultaneous learning and prediction. However, conflicts in parameter optimization among these tasks can reduce its flexibility. YOLOv8 adopts a decoupling approach, separating the object detection task into regression and classification [35]. This separation minimizes parameter interference, enhances learning efficiency, and allows for more precise adjustments in the learning process of each subtask. By eliminating the confidence detection task, YOLOv8 simplifies the model’s output and reduces the number of training parameters, potentially increasing the running speed and inference efficiency. The model applies distinct loss functions and optimization strategies for regression and classification: the regression task combines the DFL function [36] and the CIoU Loss function, while the classification task employs the BCE Loss function. Integrating three distinct loss functions facilitates quicker, more precise target localization and sustains high performance in complex scenarios with dense targets. Additionally, YOLOv8 embraces an Anchor-Free design, which eliminates reliance on dataset-specific prior knowledge and mitigates issues stemming from inaccuracies in such knowledge. The direct prediction approach better captures object contours, enhancing detection performance, reducing inference times, and improving generalization capabilities.

Other minor enhancements include disabling Mosaic data augmentation in the final 10 epochs of training [37], which has been shown to improve accuracy. In the neck of the network, removing the convolutional structure in the up-sampling stage of the PAN-FPN reduces computational complexity and speeds up inference. Unlike YOLOv5, which uses a focus structure for initial down-sampling to convert spatial information into channel information, YOLOv8 has demonstrated improved performance by replacing this structure with direct convolution. Overall, YOLOv8 exhibits superior performance and efficiency in handling complex detection tasks.

## 3. Materials and Methods

### 3.1. GTR Conversion

The YOLOv8 architecture is optimized for processing three-channel RGB images, utilizing color information to boost object recognition capabilities. In contrast, the dataset includes SAR images, which are single-channel grayscale images that offer less feature information compared to three-channel color images. When processing SAR images, a pseudo-RGB image is created by duplicating the grayscale image across all three channels. While this approach satisfies the input requirements of YOLOv8, it partially reduces detection performance. To overcome this limitation, this paper proposes a novel method for converting grayscale images into RGB images by incorporating scattering characteristics. Specifically, the original SAR image is assigned to the blue (B) channel, an image highlighting peak features extracted via a corner detection algorithm is mapped to the red (R) channel, and an image processed using a denoising filter is mapped to the green (G) channel. These channels are combined to form a new RGB image that enhances detection accuracy.

#### 3.1.1. Corner Detection Algorithm

**Figure 3:**
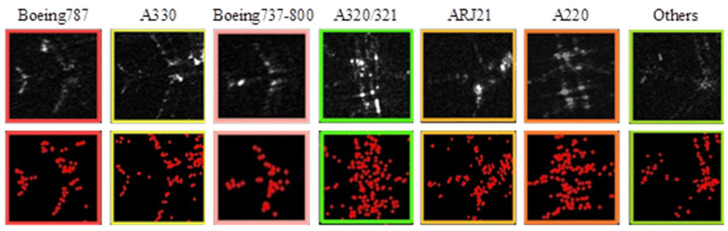
The peak features extracted using the Shi–Tomasi detection algorithm. The original aircraft target SAR images (**first row**) and the peak feature images extracted using the Shi–Tomasi detection algorithm (**second row**).

SAR images are discrete and typically of poor imaging quality. They consist of multiple irregular, bright spots known as strong scattering points, which carry crucial semantic information for identifying aircraft types. An aircraft’s structure includes the nose, fuselage, wings, and tail, with strong scattering points in SAR images often corresponding to these components’ joints. The distribution of these points reveals the geometric characteristics of aircraft targets. Different aircraft models, which have varying fuselage lengths and distinct sizes and shapes of noses, wings, and tails, therefore, display unique characteristics in their SAR images, as illustrated in Figure 3. The corner detection algorithm extracts these strong scattering points from SAR images. Integrating these peak characteristics into the RGB image can significantly enhance the accuracy of aircraft target interpretation.

Harris and Shi–Tomasi are commonly used corner detection algorithms [38,39]. They identify corner points by detecting the grayscale changes in local image windows. We adopted the Shi–Tomasi method to extract corner features of aircraft targets. The Shi–Tomasi algorithm optimizes the corner response function in the Harris algorithm, which significantly improves the detection efficiency. The following is an explanation of the basic steps of the Shi–Tomasi algorithm. Firstly, a mathematical model is established based on the corner detection process in the actual image, as shown in Formula (1).

(1) $$E(u,v)=\sum_{\left(x,y\right)}w(x,y){\left[I(x+u,y+v)-I(x,y)\right]}^{2}$$

I(x,y) is the grayscale value of the original image, (u,v) is the offset with (x,y) as the center, w(x,y) is the weighted window function centered at (x,y), and w(x,y) uses the Gaussian window function in the paper. E(u,v) is the grayscale difference calculated based on the offset of the local window. Formula (1) performed the first-order Taylor expansion and then merged and simplified to obtain Formula (2).

(2) $$\begin{array}{l} E(u,v)\approx \sum_{\left(x,y\right)}w(x,y)(u^{2}I_{x}^{2}+2uvI_{x}I_{y}+v^{2}I_{y}^{2})\approx [u,v]M\left[\begin{array}{l} u\\ v \end{array}\right]\\ M=\sum_{\left(x,y\right)}w(x,y)\left[\begin{array}{cc} I_{x}^{2} & I_{x}I_{y}\\ I_{x}I_{y} & I_{y}^{2} \end{array}\right] \end{array}$$

$I_{x}$ and $I_{y}$ are the partial derivatives of the image in the x-axis and y-axis directions. M in the formula is the covariance matrix of the image gradient, a real symmetric matrix. After diagonalization processing, the eigenvalues $\lambda_{1}$ and $\lambda_{2}$ in the two orthogonal directions are extracted. Formula (3) shows the corner response function.

(3) $$R(x,y)=\min(\lambda_{1},\lambda_{2})$$

**Figure 4:**
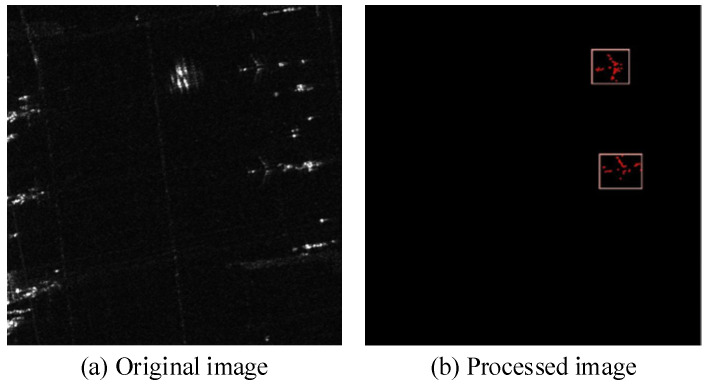
Corner detection results of aircraft targets using the Shi–Tomasi algorithm. The original SAR image (first column) and the image processed using the corner detection algorithm (in the bounding boxes).

Parameter adjustment based on experience is unnecessary; instead, smaller eigenvalues should be utilized as the scoring metric for the response function *R*(*x*, *y*). The Shi–Tomasi algorithm, compared to the Harris algorithm, demonstrates greater robustness and is particularly valuable in processing SAR images with complex noise. In the preprocessing of SAR images, the Shi–Tomasi algorithm is applied specifically within the aircraft target box to minimize the influence of background noise, as illustrated in Figure 4. However, when the trained model predicts aircraft types and lacks information about the aircraft target box, the Shi–Tomasi algorithm is applied to the entire SAR image during preprocessing.

#### 3.1.2. Multiplicative Noise-Filtering Algorithm

**Figure 5:**
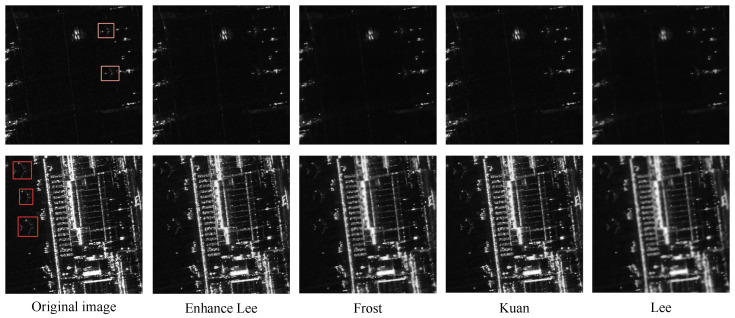
Comparison of four filtering algorithms on SAR images. The original SAR images with bounding boxes (first column) and filtered images after Enhanced Lee, Frost, Kuan, and Lee algorithms (second, third, fourth, and fifth columns).

The principle of SAR imaging is based on the coherent imaging of microwaves. Interference among electromagnetic waves generates speckle noise in SAR images, a type of multiplicative noise. This noise can obscure the detailed information of aircraft targets, cause uneven brightness across the images, and severely impact the ability to identify aircraft types. We compared and analyzed four spatial domain filtering algorithms designed to suppress speckle noise in SAR images: the Lee, Frost, Kuan, and Enhanced Lee algorithms [40,41,42,43]. Each algorithm has distinct advantages and disadvantages, but their primary objective is to smooth speckle noise while preserving the detailed information of aircraft targets. In practice, the choice of filtering algorithm is tailored to specific scenarios. We extracted two images from the dataset, and the images processed using the above four algorithms are shown in Figure 5.

It is difficult to evaluate the quality of the filtered images with the naked eye. The filtering effects are evaluated using *ENL* [44] and *ESI* [45] indicators. Section 4.3.1 will provide a detailed description of the evaluation process. Through comparative analysis, the Enhanced Lee algorithm has the best performance. We used it for filtering and denoising. The formula is as follows:

(4) $$\hat{R}(i,j)=\left\{\begin{array}{ll} \bar{I}(i,j) & C_{I}<C_{\min}\\ \bar{I}(i,j)+w(i,j)[I(i,j)-\bar{I}(i,j)] & C_{\min}<C_{I}<C_{\max}\\ I(i,j) & C_{I}>C_{\max} \end{array}\right.$$

where $\hat{R}(i,j)$ represents the filtered image, I(i,j) represents the original image, $\bar{I}(i,j)$ represents the mean value of the filtering window, and w(i,j) represents the weight coefficient. The formula is as follows:

(5) $$w(i,j)=1-\frac{C_{u}^{2}}{C_{I}^{2}}$$

where $C_{u}$ and $C_{I}$ represent the standard deviation coefficients of the filtering window and the original image, and the formula is shown as follows:

(6) $$C_{u}=\frac{\sigma_{u}}{\bar{u}}$$

(7) $$C_{I}=\frac{\sigma_{I}}{\bar{I}}$$

where $\sigma_{u}$ and $\sigma_{I}$ represent the standard deviations of the filtering window and the original image. $\bar{u}$ and $\bar{I}$ represent the mean values of the filtering window and the original image. $C_{\min}$ and $C_{\max}$ are two threshold values, and their calculation formulas are as follows:

(8) $$C_{\min}=\frac{1}{\sqrt{L}},\;C_{\max}=\sqrt{1+\frac{2}{L}}$$

where L represents the *ENL* of the original image. When ${C_{I}<C}_{\min}$, this window adopts the mean filtering algorithm. When ${C_{I}>C}_{\min}$, this window should retain the information of the original image. When ${C_{\min}<C_{I}<C}_{\max}$, this window is most affected by noise and adopts the Lee filtering algorithm.

**Figure 6:**
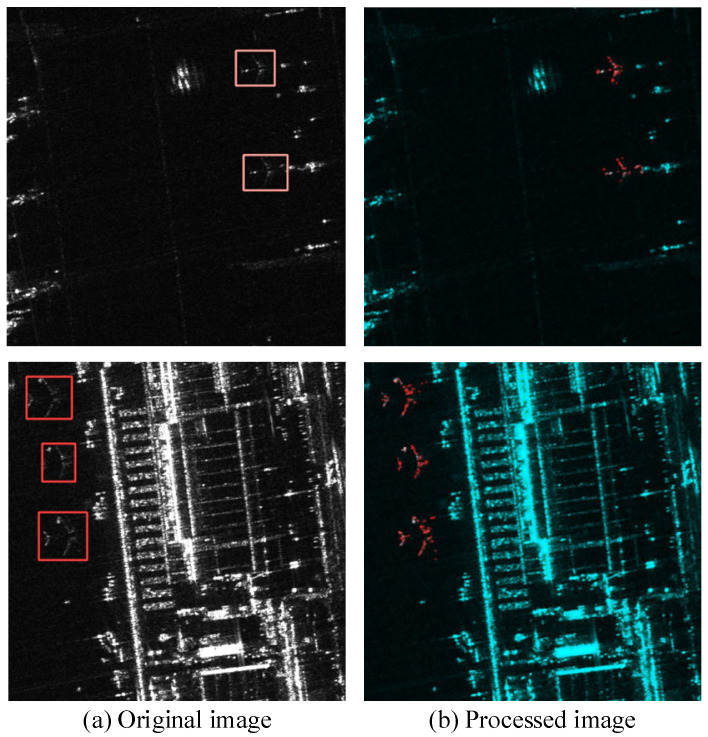
RGB images produced using the GTR method. The original SAR images with bounding boxes (first column) and the images processed using GTR method (second column).

The new RGB image is constructed by assigning the Enhanced Lee filtered image to the green channel, the original SAR grayscale image to the blue channel, and the Shi–Tomasi feature image to the red channel. This channel allocation is strategically designed to maximize the complementarity of information and enhance the network’s feature learning capability for aircraft detection. Specifically, the blue channel retains the scene’s global backscattering and intensity information from the original SAR image, providing essential contextual cues. The red channel, containing the Shi–Tomasi output, highlights corner and edge features, strengthening the network’s ability to localize structural details and contours of aircraft targets. Meanwhile, the green channel incorporates the Enhanced Lee filtered image, which improves local contrast and texture by effectively reducing speckle noise while preserving fine details, crucial for distinguishing subtle aircraft features from complex backgrounds. By distributing these complementary features across separate channels, the downstream neural network is better equipped to extract, integrate, and utilize multi-modal information during training and inference. This unified representation encourages the model to learn more robust and discriminative features, ultimately improving aircraft target detection accuracy. As illustrated in Figure 6, the newly generated RGB images form the input for subsequent training and prediction, substantially boosting overall performance in aircraft detection tasks.

### 3.2. 4SDC Detection Model

**Figure 7:**
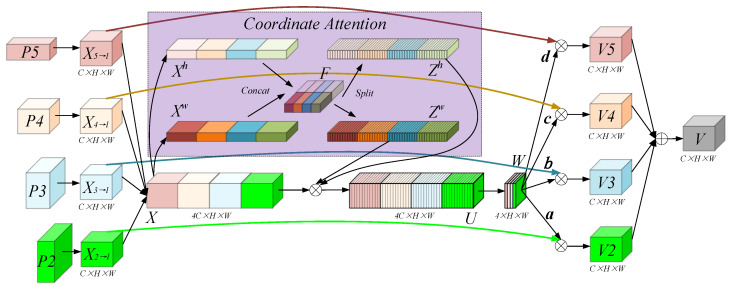
The 4SDC detection model. *P*2, *P*3, *P*4, and *P*5 are the inputs; *X*_2*→l*_, *X*_3*→l*_, *X*_4*→l*_, and *X*_5*→l*_ represent that different inputs are adjusted to a unified size at the *l* layer; *V*2, *V*3, *V*4, and *V*5 are the feature maps after adaptive fusion at the *l* layer; and *V* is the output.

The original YOLOv8 model exhibited low detection accuracy for small targets, with its detection head comprising three branches for large, medium, and small targets, respectively. To enhance detection accuracy for tiny targets, the fourth detection branch was added to the YOLOv8 model. Initially, each detection branch contributed equally to predicting a target in the original YOLOv8. However, the deeper detection branch played a more significant role for large targets, while the shallower branch was more influential for small targets. To address this, an attention mechanism was integrated into the head of the model, enabling automatic adjustment of the weights of each branch and adaptive fusion of their contributions. Prominent attention mechanisms include SENet, CBAM, and Coordinate Attention (CA) [46,47,48]. SENet focuses solely on channel dimension correlations, overlooking spatial dimensions. CBAM addresses spatial dimension correlations but is limited to local areas. The chosen CA mechanism considers spatial and channel dimension relationships and addresses long-range dependencies. In the paper, a 4SDC model was designed to enhance the detection ability for multi-scale targets, as shown in Figure 7.

*P*2, *P*3, *P*4, and *P*5 represent the four detection branches outputting from the YOLOv8 architecture. The enhancement in the algorithm involves adaptively fusing these branches at four levels. Specifically, using the *P*4 level as an example, which involves multi-scale feature fusion, we note that *l* equals 4. The feature maps of *P*2, *P*3, *P*4, and *P*5 differ in resolution and channel count. To effectively merge at the *P*4 level, adjusting the feature maps from the other levels is necessary. *P*5 performs an up-sample to change the resolution and executes 3 × 3 convolution to change the channels, and then *X*_5*→*4_ is obtained. *P*2 and *P*3 perform a down-sample to change the resolution and execute 3 × 3 convolution to change the channels, and then *X*_3*→*4_ and *X*_2*→*4_ are obtained. The sizes of the feature maps (*X*_2*→*4_, *X*_3*→*4_, *X*_4*→*4_, *X*_5*→*4_) are all *C* × *H* × *W*. They are fused in the channel dimension to obtain the feature map *X*, which has a size of 4*C* × *H* × *W*. The expression of *X* is as follows:

(9) $$X=Cat\left(\left[X_{2\to 4},X_{3\to 4},X_{4\to 4},X_{5\to 4}\right]\right)$$

Since the resolutions of feature maps at different levels are different, simple fusion will introduce conflicting information and reduce the expressive ability of feature information. We adopted the CA mechanism, which enhances the expression of channel and spatial information and suppresses interference between unrelated information. On channel dimension *C*, the feature map *X* is average pooling in the *h* and *w* directions to obtain $X^{h}$ and $X^{w}$. The calculation formulas are as follows:

(10) $$X_{c}^{h}(h)=\frac{1}{W}\sum_{0\leq i\leq W}X_{c}(h,i)$$

(11) $$X_{c}^{w}(w)=\frac{1}{H}\sum_{0\leq j\leq H}X_{c}(j,w)$$

where $X^{h}$ and $X^{w}$ are concatenated in the spatial dimension and compress the channel dimension using the full convolutional operation with a kernel size of 1 × 1, then performing the BN operation and ReLU function to obtain *F*, for which the formula is shown as follows:

(12) F=ReLU(BN(Conv(Cat[Xh,Xw])))

The *F* is split into two vectors, and the original channel dimension is recovered using the full convolutional operation with a kernel size of 1 × 1 separately. Then, a sigmoid function is performed to obtain the weight vectors $Z^{h}$ and $Z^{w}$, for which the formula is shown as follows:

(13) $$Z^{h}=Sigmoid(Conv(Split{\left(F\right)}^{h}))$$

(14) $$Z^{w}=Sigmoid(Conv(Split{\left(F\right)}^{w}))$$

The two weight vectors are multiplied by the input *X* to obtain *U*, which is shown as follows:

(15) $$U_{c}(i,j)=X_{c}(i,j)\times Z_{c}^{h}(i)\times Z_{c}^{w}(j)$$

where $U_{c}(i,j)$ and $X_{c}(i,j)$ represent the feature map of the *c*th channel of *U* and *X*. They have the same size: 4*C* × *H* × *W*. The feature map *U* can amplify the weights of important features and capture the correlations between channel and spatial information. It can also explore the long-distance dependence between input and output features to reduce the interference of redundant and conflicting information. To further capture the spatial features, *U* is convolved with a kernel size of 1 × 1 to compress the channel dimension to four to obtain *W* with a size of 4 × *H* × *W*. Then, the softmax function is performed on *W* to obtain four normalized weight matrices—*a*, *b*, *c*, and *d*—all of which are *H* × *W*. The new fused feature map *V* is obtained by multiplying the feature maps at each level with the weight matrix, which is shown as follows (*l* = 4):

(16) $$Vl=aX_{2\to l}+bX_{3\to l}+cX_{4\to l}+dX_{5\to l}$$

The weight matrices *a*, *b*, *c*, and *d* are learned and iteratively updated through network backpropagation during the model training process. These parameters adaptively adjust based on the input data to enhance aircraft target recognition in SAR images. For smaller aircraft targets, higher weights are assigned to shallow feature maps, whereas larger aircraft targets receive higher weights in deeper feature maps. This strategic allocation of weights between shallow and deep features enables more precise target recognition. Additionally, more contextual information is aggregated across feature layers with varying receptive fields, fully leveraging the multi-dimensional features of different depth layers. The resulting fused features combine robust semantic and rich textural details, thereby improving the detection and discrimination of small targets.

### 3.3. C2f-SWTran Feature Extraction Module

**Figure 8:**
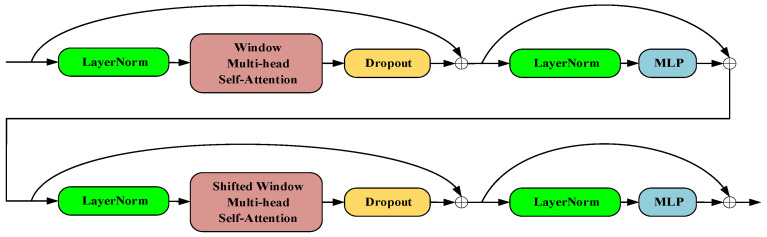
Structural diagram of the Swin Transformer block. The model applies window-based and shifted window multi-head self-attention, combined with normalization, dropout, and MLP, to efficiently extract hierarchical features.

The backbone of YOLOv8 utilizes multiple convolutional layers and residual connections, which are effective for capturing local and hierarchical information from images. However, this convolutional architecture is limited in modeling long-range feature dependencies, as it relies on relatively small receptive fields and fixed-scale feature extraction. This limitation reduces the model’s robustness, especially in complex SAR scenarios. To address these challenges, we introduce the Swin Transformer architecture, which excels at capturing global feature dependencies through its shifted window and hierarchical structure. This design enables the effective modeling of both local and global representations, which is essential for complex visual tasks. Moreover, compared to standard global self-attention mechanisms, the Swin Transformer significantly reduces computational complexity, making it more suitable for large-scale vision applications. By integrating the strengths of both traditional convolutional structures and the Swin Transformer, we achieve complementary feature extraction. Based on this motivation, we developed the C2f-SWTran module to enhance detection accuracy. Figure 8 illustrates the architecture of the Swin Transformer.

The Swin Transformer block comprises two sub-layers that function in tandem. Its core components, W-MSA and SW-MSA, enhance the traditional Transformer architecture. W-MSA divides the full feature map into multiple non-overlapping windows and conducts self-attention independently within each window, reducing computational demands and enhancing efficiency. However, this approach initially isolated information transfer between windows. To address this limitation, SW-MSA was introduced to facilitate information flow between adjacent windows, thereby improving the model’s grasp of global information in SAR images. The Swin Transformer structure significantly boosts both performance and efficiency, making it particularly effective for computer vision detection tasks in complex environments. In the YOLOv8 architecture, Swin Transformer acts as a plug-and-play module following the C2f module, and together with C2f, it forms the integrated C2f-SWTran module.

### 3.4. Improved YOLOv8 Structure

**Figure 9:**
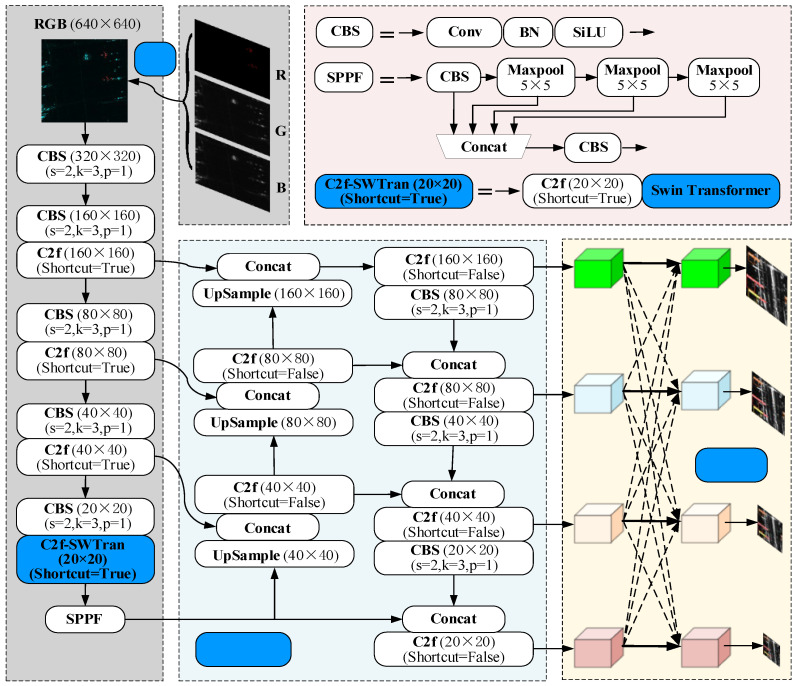
Overview of the improved YOLOv8 architecture. The model integrates the GTR, C2f-SWTran, and 4SDC modules to enhance feature extraction and multi-scale fusion, improving detection accuracy for SAR targets.

YOLOv8, known for its fast inference speed and high-precision detection, serves as the baseline for our study. We implemented three enhancements to improve the detection accuracy of aircraft targets. First, we transformed grayscale images in the dataset into RGB images using the Shi–Tomasi algorithm and Enhanced Lee filtering, which enhances the feature representation ability of SAR images and makes them more compatible with the requirement of the YOLOv8 model for inputting three-channel color images. Second, we developed the 4SDC module to improve the detection accuracy of multi-scale targets, especially for small targets, where the improvement in detection ability is more evident. Third, we integrated the Swin Transformer into YOLOv8′s backbone to enhance the detection accuracy. Figure 9 illustrates the improved YOLOv8 model.

## 4. Results

To validate the effectiveness of our improvement strategies, we conducted a series of ablation studies. These studies are detailed in three main sections: an overview of the IS-PRS-SAR-Aircraft and SAR-Aircraft-1.0 datasets, a description of the experimental setup, and a comparative analysis of the methods and results.

### 4.1. ISPRS-SAR-Aircraft Dataset and SAR-AIRcraft-1.0 Dataset

**Figure 10:**
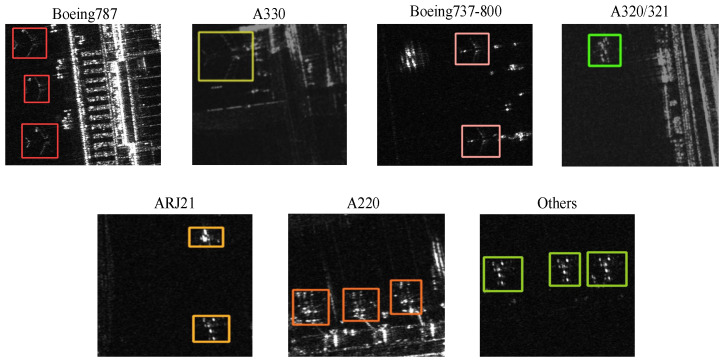
Examples of seven aircraft categories in SAR images. Each image shows representative samples with colored boxes marking different aircraft types, illustrating the dataset’s diversity and annotation quality.

Both datasets are constructed based on the images from the domestic Gaofen-3 satellite, covering the image data of multiple civil airports at different periods. Each dataset contains seven aircraft categories. We have carefully selected seven representative images from the two datasets (Figure 10). Each image provides detailed category information. However, the presence of non-target elements such as terminal buildings and baggage trolleys, along with coherent speckle noise, contributes to the complexity of the SAR images. These images are characterized by complex scenes, diverse noises, and multiple scales, heightening the challenge of recognizing aircraft types.

**Figure 11:**
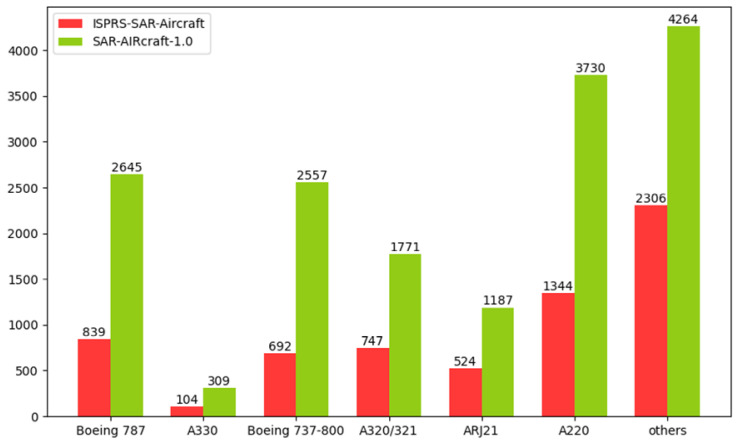
The quantity of each aircraft type in the two datasets.

The ISPRS-SAR-Aircraft dataset contains 2000 SAR images and 6556 aircraft instances. The SAR-AIRcraft-1.0 dataset contains 4368 SAR images and 16,463 aircraft instances. The SAR images in the training, validation, and test sets are distributed according to an 8:1:1 ratio, and the processing methods of the two datasets are consistent. The number of each type of aircraft is illustrated in Figure 11.

**Figure 12:**
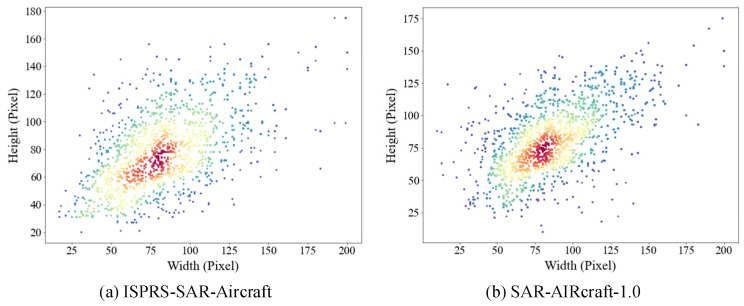
Size distribution of target boxes in both SAR datasets. Different colors represent the different distribution densities of target box sizes.

The sizes of the aircraft target boxes in the two datasets vary significantly, and there is a relatively large number of small targets. We classify anchor boxes smaller than 60 pixels as small targets. The ISPRS-SAR-Aircraft dataset contains 6556 aircraft instances, among which 1956 are small targets, and their distribution is shown in Figure 12a. The SAR-AIRcraft-1.0 dataset contains 16,463 aircraft instances, among which 3874 are small targets, and their distribution is illustrated in Figure 12b.

### 4.2. The Configuration of Our YOLOv8 Experimental Environment

All experiments were conducted on a 64-bit Windows 11 system equipped with an NVIDIA GeForce RTX 4090 GPU, utilizing CUDA 12.4 for acceleration. The network was implemented in PyTorch 2.5.1 with Python 3.12.8. Hyperparameter configurations are summarized in Table 1. Our choices were informed by both best practices in the literature and preliminary validation experiments. The learning rate (0.01) and optimizer (SGD) were selected for their reliable convergence and robust performance in YOLO-based object detection tasks. A batch size of 32 was determined to offer an optimal balance between computational efficiency and detection accuracy. Data augmentation parameters—including Mosaic, Flipud, Fliplr, and Scale—were tuned through grid search within reasonable ranges, with final values selected based on peak mean Average Precision (*mAP*) on the validation set while mitigating overfitting.

### 4.3. Experimental Results

#### 4.3.1. Experimental Analysis of the Enhanced Lee Algorithm

In the paper, the coherent speckle-noise-filtering algorithms of Lee, Frost, Kuan, and Enhanced Lee were adopted for SAR images. Different algorithms may be suitable for various application scenarios. Evaluating the quality of these filtering algorithms through human observation is challenging. Therefore, we employed two indicators, *ENL* and *ESI*, to assess the effectiveness of the filtering algorithms. *ENL* serves as a measure for assessing the noise level and texture smoothness in SAR images. The intensity of the noise is evaluated using *ENL*. The better the performance of the filtering algorithm in noise suppression, the higher the *ENL* value. The formula of *ENL* is as follows:

(17) $$\mathrm{ENL}=\frac{\mu^{2}}{\sigma^{2}}$$

where *µ* represents the mean value of the SAR image, and *σ* represents the variance of the SAR image. *ESI* is an indicator for evaluating the edge information preservation ability of different SAR image-filtering algorithms. *ESI* can measure whether the image-filtering algorithm can effectively retain the edge and detail information while removing noise. The closer the *ESI* value is to 1, the more effectively the filtering algorithm retains edge and detail information in the SAR image. The formula for *ESI* is shown below:

(18) $$ESI=\frac{\sum_{i}\sum_{j}\left[\left|\hat{F}(i+1,j)-\hat{F}(i,j)\right|+\left|\hat{F}(i,j+1)-\hat{F}(i,j)\right|\right]}{\sum_{i}\sum_{j}\left[\left|F(i+1,j)-F(i,j)\right|+\left|F(i,j+1)-F(i,j)\right|\right]}$$

where F(i,j) represents the pixel’s grayscale value corresponding to position (i,j) in the SAR image before filtering. $\hat{F}(i,j)$ represents the pixel’s grayscale value corresponding to position (i,j) in the SAR image after filtering. *ENL* and *ESI* are usually mutually constrained. Increasing the *ENL* will reduce the *ESI*, and increasing the *ESI* will reduce the *ENL*. In SAR image recognition, edge information includes the crucial feature. If the edge information is weakened seriously, it will significantly affect the subsequent recognition of aircraft targets. Therefore, algorithms with higher *ESI* values are preferentially selected. We conducted experiments using the four filtering algorithms on all SAR images in the dataset. The average *ENL* and *ESI* values were calculated and are presented in Table 2:

In SAR images, the edge information of the aircraft target, processed using the Enhanced Lee algorithm, is optimally preserved with an *ESI* value of 0.883. This algorithm adjusts its response based on the pixel distribution within different filtering windows: it suppresses intense noise and ignores mild noise, effectively reducing noise in SAR images. For this study, we utilized the Enhanced Lee algorithm for the filtering process.

#### 4.3.2. Experimental Analysis of GTR

As described in Section 3.1, we utilized the Shi–Tomasi corner detection algorithm and the Enhanced Lee noise-filtering algorithm to transform grayscale images in the SAR dataset into RGB images, enriching the information content and creating the RGB dataset. We adopt two assessment parameters: *AP* and *mAP*. *AP* is an indicator for measuring the performance of single-type aircraft target detection. The higher its value, the better the detection performance of the algorithm. Its formula is as follows:

(19) $$AP=\int_{0}^{1}P(R)dR$$

where *AP* represents the area enclosed by the *P-R* curve and the coordinate axes, and the calculation formulas for *P* and *R* are as follows:

(20) $$R=\frac{TP}{TP+FN}$$

(21) $$P=\frac{TP}{TP+FP}$$

where *P* and *R* are determined by *TP*, *FP,* and *FN*. The first character indicates whether the prediction is correct. *T* represents correct prediction. *F* represents incorrect prediction. The second character represents the actual predicted result. *P* represents the positive class. *N* indicates the negative class. *TP* represents the number of aircraft targets predicted correctly. *FP* represents the number of false alarms for aircraft targets. *FN* represents the number of aircraft targets missed in the detection. *mAP* is the average value of *AP* for all types of aircraft. The higher its value, the better the comprehensive detection performance of the algorithm. The dataset contains a total of seven types of aircraft targets, and its formula is shown as follows:

(22) $$mAP=\frac{1}{7}\sum_{i=1}^{7}AP_{i}$$

where *mAP*_50_ indicates that the IoU threshold is 0.5. *mAP*_50~95_ indicates that the IoU threshold is between 0.5 and 0.95, with an interval of 0.05 to obtain the average of 10 *mAP*s. To evaluate the effectiveness of the GTR, we conducted the following ablation experiments:

As shown in Table 3 below, we performed ablation experiments on two datasets using YOLOv8n and YOLOv8l models as baselines. “×” indicates the non-use, while “√” signifies use. For the dataset ISPRS-SAR-Aircraft, integrating the GTR method improved the *mAP*_50~95_ of YOLOv8n by 1.3% and the *mAP*_50~95_ of YOLOv8l by 1.2%. For the dataset SAR-AIRcraft-1.0, integrating the GTR method improved the *mAP*_50~95_ of YOLOv8n by 1.9% and the *mAP*_50~95_ of YOLOv8l by 2.1%. This experiment demonstrates that the GTR method effectively enriches the features of SAR images, thereby improving the detection accuracy of aircraft targets.

#### 4.3.3. Experimental Analysis of 4SDC Method

The 4SDC module extends the original three detection branches of YOLOv8 to include a fourth detection branch, which is integrated after the second feature layer of the backbone structure primarily to enhance detection capabilities for small targets. The CA mechanism is incorporated into the detection head, enabling adaptive adjustment of the contribution degrees of different detection branches when detecting aircraft targets, thereby improving the overall detection capabilities. To evaluate the performance of the 4SDC module, we conducted ablation studies. The experimental results are shown in Table 4.

We established two additional validation sets: the small-target validation set and the large-target validation set. Images containing small aircraft targets (less than 60 × 60 pixels) are classified into the small-target validation set, while all other images are assigned to the large-target validation set. In the ISPRS-SAR-Aircraft dataset, the small-target set includes 397 SAR images, and the large-target set comprises 1603 SAR images. Similarly, in the SAR-Aircraft-1.0 dataset, the small-target set contains 866 images, and the large-target set includes 3502 images. *S-aircraft* refers to the small-target validation set, and *L-aircraft* refers to the large-target validation set. For the dataset ISPRS-SAR-Aircraft, the integration of 4SDC improved the *mAP*_50~95_ of YOLOv8n with GTR by 0.6%, and the *mAP*_50~95_ of *S-aircraft* was improved by 0.9%. The integration of 4SDC improved the *mAP*_50~95_ of YOLOv8l with GTR by 0.5% and the *mAP*_50~95_ of *S-aircraft* by 0.8%. For the dataset SAR-AIRcraft-1.0, the integration of 4SDC improved the *mAP*_50~95_ of YOLOv8n with GTR by 0.7%, and the *mAP*_50~95_ of *S-aircraft* was improved by 1.6%. The integration of 4SDC improved the *mAP*_50~95_ of YOLOv8l with GTR by 0.6% and the *mAP*_50~95_ of *S-aircraft* by 1.4%. The ablation experiments confirmed that the 4SDC structure enhances the detection accuracy of aircraft targets, particularly for small targets. The prediction results are displayed in the figure below.

**Figure 13:**
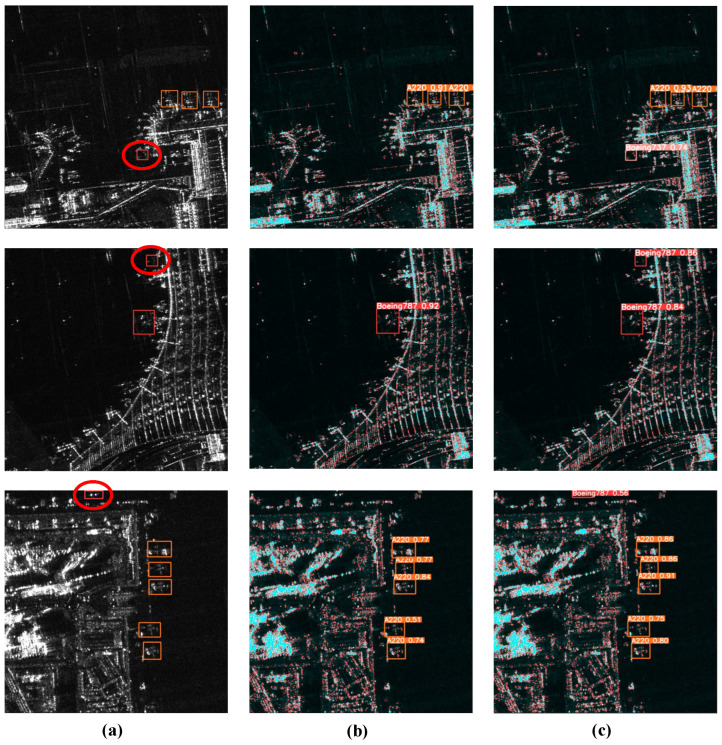
Comparison of results for 4SDC. (**a**) The original SAR images with bounding boxes. (**b**) Recognition results of YOLOv8l with GTR. (**c**) Recognition results of YOLOv8l with GTR and 4SDC.

Figure 13 shows the effect diagram of aircraft target recognition after introducing the 4SDC structure. Rectangular boxes of different colors in the figure represent different types of aircraft targets. In Figure 13a, the aircraft marked with red circles are small targets. Figure 13b illustrates that YOLOv8l, when combined with the GTR algorithm, exhibits more missed detections, adversely affecting the overall detection accuracy of SAR images featuring aircraft targets. Incorporating 4SDC enhances the algorithm’s detection performance for small targets, as demonstrated in Figure 13c.

#### 4.3.4. Experimental Analysis of C2f-SWTran Module

The C2f-SWTran is a plug-and-play module that, when applied to the YOLOv8 backbone, enhances the capture of global features in SAR images. This module incorporates a self-attention mechanism, which can impact inference speed due to its large parameter count. Consequently, only one C2f-SWTran module was incorporated into the backbone structure. The YOLOv8 backbone comprises five feature layers of varying image sizes. Applying the C2f-SWTran module to the shallow feature maps would increase parameter consumption. Therefore, we opted to integrate this module into the fifth-layer feature map, which has the smallest image size, balancing accuracy and efficiency. Subsequent ablation studies were conducted to evaluate this approach.

For the ISPRS-SAR-Aircraft dataset, the C2f-SWTran module achieves enhancements of 0.5% and 0.6% in the *mAP*_50~95_ for YOLOv8n and YOLOv8l (with GTR + 4SDC), respectively. C2f-SWTran still achieves 0.6% and 0.7% enhancements in the *mAP*_50~95_ for YOLOv8n and YOLOv8l (without GTR + 4SDC), respectively. For the SAR-AIRcraft-1.0 dataset, the C2f-SWTran module achieves enhancements of 0.6% and 0.7% in the *mAP*_50~95_ for YOLOv8n and YOLOv8l (with GTR + 4SDC), respectively. C2f-SWTran still achieves 0.7% and 0.8% enhancements in the *mAP*_50~95_ for YOLOv8n and YOLOv8l (without GTR + 4SDC), respectively. Regardless of whether the YOLOv8 algorithm is configured with the GTR + 4SDC module, the degree of improvement in the detection accuracy by the C2f-SWTran module remains the same and is not affected by the GTR + 4SDC module, indicating that the C2f-SWTran module and the GTR + 4SDC module are independent of each other. By applying the same analytical method to both Table 4 and Table 5, similar conclusions can be drawn: the 4SDC module and the GTR + C2f-SWTran module are independent of each other. The three key modules—GTR, 4SDC, and C2f-SWTran—are designed to be complementary and independent. The improvement in detection accuracy by each module does not affect other modules, each addressing different aspects of the detection task. Their combined use leads to a significant overall performance improvement.

*PARAM* reflects the size of the model parameters. *GFLOPs* reflect the computational complexity of the model. From the experimental data, it is found that YOLOv8n and YOLOv8l with C2f-SWTran have a *PARAM* that is 0.24 MB and 1.1 MB larger, respectively, and *GFLOPs* that are 1.7 G and 10.6 G larger, respectively, compared to the original YOLOv8n and YOLOv8l. YOLOv8n and YOLOv8l with GTR + 4SDC + C2f-SWTran have a *PARAM* that is 0.25 MB and 1.1 MB larger, respectively, and *GFLOPs* that are 1.7 G and 10.6 G larger, respectively, compared to the YOLOv8n and YOLOv8l with GTR + 4SDC. The results of the ablation experiment show that after integrating the C2f-SWTran module into the fifth-layer feature map of the YOLOv8 backbone network, the overall model experiences only a slight increase in the number of parameters and computational complexity. However, there is a certain degree of improvement in the detection accuracy of aircraft targets. Therefore, the added computational cost is reasonable relative to the performance boost, and inference remains efficient for practical use.

#### 4.3.5. Comparative Analysis of Improved Algorithms with Other Algorithms

To further demonstrate the effectiveness and superiority of our proposed method, we conducted additional experiments comparing it with state-of-the-art non-YOLO models, including Faster R-CNN, SSD, SKG-DDT [49], and EBPA2N [25]. The results are presented in Table 6 below.

Our proposed method—based on YOLOv8l with the integration of GTR, 4SDC, and C2f-SWTran modules (last row for each dataset)—achieves the highest detection accuracy across both datasets. Specifically, (1) on the ISPRS-SAR-Aircraft dataset, our method achieves an *mAP*_50_ of 0.932 and an *mAP*_50~95_ of 0.742, outperforming all other models. Notably, compared to non-YOLO methods, such as Faster R-CNN (*mAP*_50_: 0.859) and SSD (*mAP*_50_: 0.805), our approach improves the *mAP*_50_ by over 7.3% and 12.7%. (2) On the SAR-AIRcraft-1.0 dataset, our method achieves an *mAP*_50_ of 0.929 and an *mAP*_50~95_ of 0.683. This again exceeds both traditional and recent non-YOLO approaches, such as Faster R-CNN (*mAP*_50_: 0.838), SSD (*mAP*_50_: 0.795), SKG-DDT (*mAP*_50_: 0.892), YOLOv7 (*mAP*_50_: 0.880), and EBP-A2N (*mAP*_50_: 0.913), where our approach improves the *mAP*_50_ by over 9.1%, 13.4%, 3.7%, 4.9%, and 1.6%. In addition, for the ISPRS-SAR-Aircraft dataset, the improved YOLOv8n improves the *mAP*_50~95_ by 2.4% compared to the original YOLOv8n. The improved YOLOv8l algorithm improves the *mAP*_50~95_ by 2.3% compared to the original YOLOv8l. For the SAR-AIRcraft-1.0 dataset, the improved YOLOv8n improves the *mAP*_50~95_ by 3.2% compared to the original YOLOv8n. The improved YOLOv8l improves the *mAP*_50~95_ by 3.4% compared to the original YOLOv8l. The performance gap further demonstrates the effectiveness of our proposed modules in improving SAR aircraft detection.

**Figure 14:**
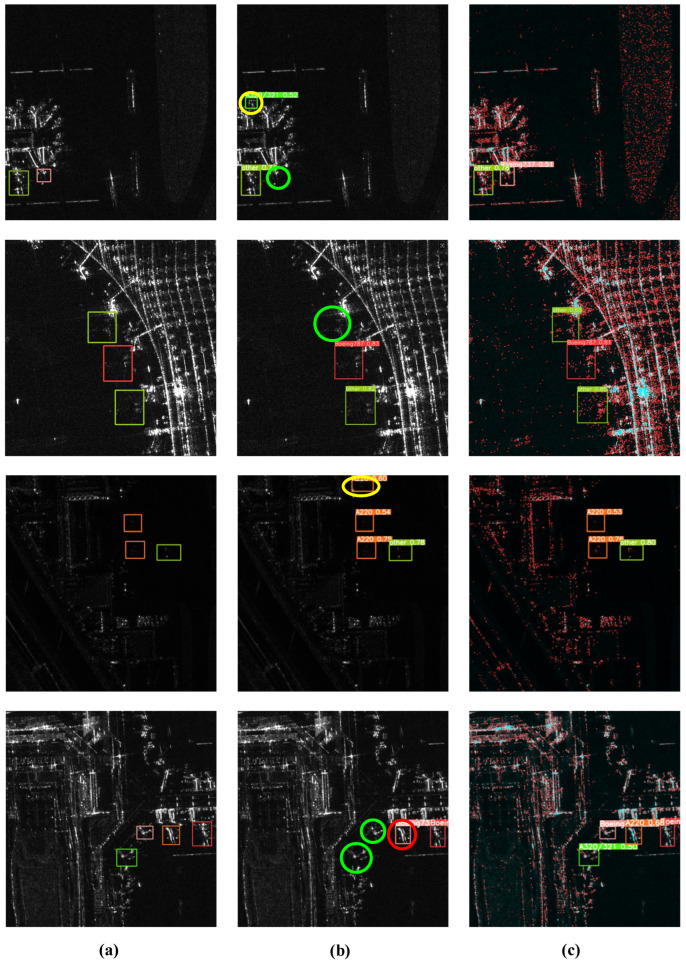
Comparison of the results. (**a**) The original SAR images with bounding boxes. (**b**) Recognition results of the original YOLOv8l. (**c**) Recognition results of our improved YOLOv8l.

Experimental analysis suggests that the original YOLOv8 model frequently encounters issues with missed detections, false detections, and false alarms for aircraft targets. As depicted in Figure 14b, green circles indicate missed detections, red circles denote false detections, and yellow circles signify false alarms of aircraft targets. Integrating the three improvement methods designed in this study into YOLOv8 has significantly enhanced the accuracy by reducing the rates of missed detections, false detections, and false alarms, as demonstrated in Figure 14c.

#### 4.3.6. Discussions About Cross-Dataset Adaption

Although the method designed in this paper is applied to the recognition of aircraft targets in SAR images, it contributes a general-purpose solution to the SAR image target recognition field. To thoroughly verify the universality of this method, this paper introduces the MSAR-1.0 dataset for further research [50]. This dataset contains 28,449 detection slices sourced from the Haise-1 and Gaofen-3 satellite data. The image includes four categories of targets: aircraft, oil tanks, bridges, and ships.

**Figure 15:**
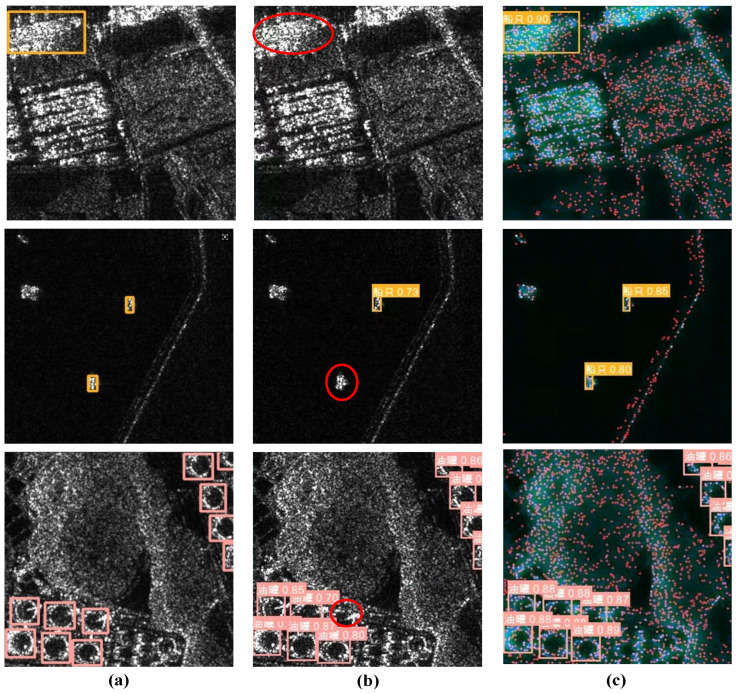
Comparison of the results. (**a**) The original SAR images with bounding boxes. (**b**) Recognition results of the original YOLOv8n. (**c**) Recognition results of our improved YOLOv8n.

In this paper, YOLOv8n is still used as the baseline. The results of the comparative analysis between the improved method and the original YOLOv8n are shown in Table 7, and the detection effects are shown in Figure 15. The original YOLOv8n detection algorithm has a relatively high number of missed detections, as shown by the red circles in Figure 15b. The improved method we proposed alleviates this problem of missed detections and improves target detection accuracy. The experiment demonstrates that the proposed method applies to recognizing targets in SAR images to a certain extent. In Figure 15, “船只” represents Ship, and “油罐” represents oil tank.

## 5. Discussion

To ensure the detection accuracy and stability of aircraft targets, we adopted the YOLOv8 model as the baseline. Due to SAR’s imaging characteristics, directly applying the YOLOv8 model to aircraft target recognition will lead to many missed detections, false detections, and false alarms. Therefore, we optimized the entire detection process. Firstly, we proposed the GTR method to convert SAR grayscale images into RGB images by integrating the corner features of aircraft targets and the speckle-noise suppression algorithm. GTR can improve the detection accuracy of aircraft targets. It is effective not only for the YOLOv8 model but also for other deep-learning models. Secondly, we introduced a small target detection branch and integrated the CA mechanism into the detection head, effectively enhancing the detection accuracy of multi-scale targets, particularly for small targets. Finally, regarding the scattering interference in complex airport scenes, we designed the C2f-SWTran module, which considers the long-range dependence of features to further improve the detection accuracy of aircraft targets. The experiments prove that the improved YOLOv8 model can achieve optimal detection accuracy.

Due to the complexity of airport scenes, numerous interfering factors exist, such as terminal buildings, aircraft tractors, and baggage carts. Their strong scattering characteristics can all affect the identification of aircraft targets. The structure of the aircraft is also relatively complex. Different components exhibit diverse and complex scattering characteristics and are also angle sensitive. All these characteristics increase the difficulty of identifying aircraft targets. It is not easy to significantly improve the detection accuracy of aircraft targets relying solely on single-source SAR signals. To further enhance the detection accuracy, our subsequent research direction is to fuse optical images with SAR images to provide more abundant contextual information. The color, texture, and other details in optical images can help identify aircraft targets, leading to more accurate identification of aircraft types. In addition, although the GTR method proposed in the paper significantly improves the recognition accuracy of aircraft targets by fusing multi-dimensional features, it also introduces an additional SAR image preprocessing step. The entire process of aircraft target recognition is carried out in stages, making the processing procedure rather cumbersome. To address this issue, our subsequent research will explore integrating the multi-dimensional feature fusion strategy into the architecture of deep convolutional models to achieve an end-to-end network structure design. This will simplify the existing complex process and enhance the operational efficiency of the algorithm.

## Figures and Tables

**Table 1:** The configurations of hyperparameters.

Parameter	Configuration
Epochs	500
Batch size	32
Learning rate	0.01
Optimizer	SGD
Mosaic	1.0
Flipud and Fliplr	0, 0.5
Scale	0.5

**Table 2:** *ENL* and *ESI* values for different filter algorithms.

Parameter	*ENL*	*ESI*
No	0.962	1.000
Lee	1.644	0.302
Frost	1.305	0.497
Kuan	1.134	0.830
Enhanced Lee	1.086	0.883

**Table 3:** Comparative experiment based on the GTR method.

Dataset	Model	GTR	*mAP*_50_	*mAP*_50~95_
ISPRS-SAR-Aircraft	YOLOv8n	×	0.915	0.699
	YOLOv8n	√	0.923	0.712
	YOLOv8l	×	0.920	0.719
	YOLOv8l	√	0.927	0.731
SAR-AIRcraft-1.0	YOLOv8n	×	0.896	0.629
	YOLOv8n	√	0.905	0.648
	YOLOv8l	×	0.908	0.649
	YOLOv8l	√	0.919	0.670

**Table 4:** Comparative experiment based on the 4SDC method.

Dataset	Model	GTR	4SDC	*mAP*_50_	*mAP*_50~95_	*S-aircraft* *mAP*_50~95_	*L-aircraft* *mAP*_50~95_
ISPRS-SAR-Aircraft	YOLOv8n	√	×	0.923	0.712	0.684	0.726
	YOLOv8n	×	√	0.921	0.706	0.691	0.721
	YOLOv8n	√	√	0.926	0.718	0.693	0.728
	YOLOv8l	√	×	0.927	0.731	0.720	0.736
	YOLOv8l	×	√	0.923	0.725	0.721	0.725
	YOLOv8l	√	√	0.929	0.736	0.728	0.737
SAR-AIRcraft-1.0	YOLOv8n	√	×	0.905	0.648	0.610	0.657
	YOLOv8n	×	√	0.901	0.637	0.618	0.643
	YOLOv8n	√	√	0.909	0.655	0.626	0.66
	YOLOv8l	√	×	0.919	0.670	0.642	0.678
	YOLOv8l	×	√	0.913	0.656	0.644	0.663
	YOLOv8l	√	√	0.922	0.676	0.656	0.680

**Table 5:** Comparative experiment based on the C2f-SWTran module.

Dataset	Model	GTR	4SDC	C2f SWTran	*mAP*_50_	*mAP*_50~95_	*PARAM* (MB)	*GFLOPs* (G)
ISPRS-SAR-Aircraft	YOLOv8n	×	×	×	0.915	0.699	3.01	8.7
	YOLOv8n	×	×	√	0.921	0.705	3.25	10.4
	YOLOv8n	√	√	×	0.926	0.718	4.76	10.9
	YOLOv8n	√	×	√	0.925	0.717	3.25	10.4
	YOLOv8n	×	√	√	0.923	0.711	5.01	12.6
	YOLOv8n	√	√	√	0.930	0.723	5.01	12.6
	YOLOv8l	×	×	×	0.920	0.719	43.6	165.7
	YOLOv8l	×	×	√	0.925	0.726	44.7	176.3
	YOLOv8l	√	√	×	0.929	0.736	53.4	177.5
	YOLOv8l	√	×	√	0.928	0.736	44.7	176.3
	YOLOv8l	×	√	√	0.927	0.732	54.5	188.1
	YOLOv8l	√	√	√	0.932	0.742	54.5	188.1
SAR-AIRcraft-1.0	YOLOv8n	×	×	×	0.896	0.629	3.01	8.7
	YOLOv8n	×	×	√	0.903	0.636	3.25	10.4
	YOLOv8n	√	√	×	0.909	0.655	4.76	10.9
	YOLOv8n	√	×	√	0.907	0.654	3.25	10.4
	YOLOv8n	×	√	√	0.906	0.650	5.01	12.6
	YOLOv8n	√	√	√	0.915	0.661	5.01	12.6
	YOLOv8l	×	×	×	0.908	0.649	43.6	165.7
	YOLOv8l	×	×	√	0.916	0.657	44.7	176.3
	YOLOv8l	√	√	×	0.922	0.676	53.4	177.5
	YOLOv8l	√	×	√	0.921	0.676	44.7	176.3
	YOLOv8l	×	√	√	0.918	0.666	54.5	188.1
	YOLOv8l	√	√	√	0.929	0.683	54.5	188.1

**Table 6:** Comparative experiment with other algorithms.

Dataset	Model	GTR	4SDC	C2f-SWTran	*mAP*_50_	*mAP*_50~95_
ISPRS-SAR-Aircraft	Faster R-CNN	×	×	×	0.859	0.557
	Faster R-CNN	√	×	×	0.876	0.583
	SSD	×	×	×	0.805	0.518
	SSD	√	×	×	0.829	0.558
	YOLOv3	×	×	×	0.866	0.605
	YOLOv5s	×	×	×	0.873	0.635
	YOLOv8n	×	×	×	0.915	0.699
	YOLOv8n	√	√	√	0.930	0.723
	YOLOv8l	×	×	×	0.920	0.719
	YOLOv8l	√	√	√	0.932	0.742
SAR-AIRcraft-1.0	Faster R-CNN	×	×	×	0.838	0.526
	Faster R-CNN	√	×	×	0.859	0.550
	SSD	×	×	×	0.795	0.500
	SSD	√	×	×	0.816	0.529
	YOLOv3	×	×	×	0.858	0.588
	YOLOv5s	×	×	×	0.865	0.609
	SKG-DDT	×	×	×	0.892	0.637
	YOLOv7	×	×	×	0.880	0.625
	EBPA2N	×	×	×	0.913	NA
	YOLOv8n	×	×	×	0.896	0.629
	YOLOv8n	√	√	√	0.915	0.661
	YOLOv8l	×	×	×	0.908	0.649
	YOLOv8l	√	√	√	0.929	0.683

**Table 7:** Comparative experiment with original YOLOv8.

Dataset	Model	Aircraft	Oil Tank	Bridge	Ship	*mAP*_50_	*mAP*_50~95_
MSAR-1.0	YOLOv8n	0.739	0.939	0.909	0.961	0.887	0.661
	Ours	0.784	0.948	0.916	0.976	0.906	0.699

## Data Availability

The ISPRS-SAR-Aircraft dataset was provided by the 2021 Gaofen challenge on Automated High-Resolution Earth Observation Image Interpretation (available online: http://gaofen-challenge.com; accessed on 1 October 2021). The SAR-Aircraft-1.0 dataset is provided by the paper “SAR-Aircraft-1.0: High-Resolution SAR Aircraft Detection and Recognition Dataset” published in the 4th 2023 issue of the *Journal of Radars* (available online: https://radars.ac.cn/article/doi/10.12000/JR23043?viewType=HTML; accessed on 2 February 2024).
